# Evaluation of a programming algorithm for deep brain stimulation in dystonia used in a double-blind, sham-controlled multicenter study

**DOI:** 10.1186/s42466-019-0032-2

**Published:** 2019-09-24

**Authors:** Frank Steigerwald, Anna Dalal Kirsch, Andrea A. Kühn, Andreas Kupsch, Joerg Mueller, Wilhelm Eisner, Günther Deuschl, Daniela Falk, Alfons Schnitzler, Inger Marie Skogseid, Juliane Vollmer-Haase, Chi W. Ip, Volker Tronnier, Jan Vesper, Markus Naumann, Jens Volkmann, for the DBS study group for dystonia, Andreas Kupsch, Bianca Müller, Gerd-Helge Schneider, Thomas Trottenberg, Alfons Schnitzler, Volker Sturm, Lars Timmermann, Jürgen Voges, Lars Wojtecki, Guido Nikkah, Markus O. Pinsker, Thomas Prokop, Jan Vesper, Manja Kloss, Martin Krause, Volker Tronnier, Wilhelm Eisner, Thomas Fiegele, Joerg Mueller, Sasha Hering, Werner Poewe, Günther Deuschl, Jan Herzog, Maximilian M. Mehdorn, Marcus O. Pinsker, Monika Pötter, Frank Steigerwald, Jens Volkmann, Hans-Werner Boothe, Angela Brentrup, Juliane Vollmer-Haase, Geir Ketil Roeste, Inger Marie Skogseid, Reiner Benecke, Jan-Uwe Müller, Matthias Wittstock, Alexander Wolters, Joseph Classen, Markus Naumann, Alex Schramm

**Affiliations:** 1 Department of Neurology, University Hospital Würzburg, Würzburg, Germany; 2 Department of Neurology and Neurological Critical Care, Rhön-Klinikum, Bad Neustadt, Germany; 3 Department of Neurology, Campus Mitte, Charité - Universitätsmedizin Berlin, Berlin, Germany; 4 Neurology Moves, Movement Disorder Center Berlin, Berlin, Germany; 5 Department of Neurology, Vivantes Hospital Berlin Spandau, Berlin, Germany; 6 Department of Neurology, Medical University of Innsbruck, Innsbruck, Austria; 7 Department of Neurosurgery, Medical University of Innsbruck, Innsbruck, Austria; 8 Department of Neurology, Christian Albrechts University, Kiel, Germany; 9 Department of Neurosurgery, Christian Albrechts University, Kiel, Germany; 10 Department of Neurology and Institute of Clinical Neuroscience and Medical Psychology, Heinrich Heine University, Düsseldorf, Germany; 11 Department of Neurology, Oslo University Hospital, Oslo, Norway; 12 MVZ, Evang. Kliniken, Gelsenkirchen, Germany; 13 Department of Neurosurgery, University of Lübeck, Lübeck, Germany; 14 Department of Functional Neurosurgery and Stereotaxy, Albert Ludwig University Freiburg, Freiburg, Germany; 15 Department of Functional Neurosurgery and Stereotaxy, Heinrich Heine University Düsseldorf, Düsseldorf, Germany; 16 Department of Neurology, Klinikum Augsburg, Augsburg, Germany

**Keywords:** Deep brain stimulation, Programming algorithm, Dystonia, Pallidum, Long-term outcome

## Abstract

**Background:**

Programming deep brain stimulation in dystonia is difficult because of the delayed benefits and absence of evidence-based guidelines. Therefore, we evaluated the efficacy of a programming algorithm applied in a double-blind, sham-controlled multicenter study of pallidal deep brain stimulation in dystonia.

**Methods:**

A standardized monopolar review to identify the contact with the best acute antidystonic effect was applied in 40 patients, who were then programmed 0.5 V below the adverse effect threshold and maintained on these settings for at least 3 months, if tolerated. If no acute effects were observed, contact selection was based on adverse effects or anatomical criteria. Three-year follow-up data was available for 31 patients, and five-year data for 32 patients. The efficacy of the algorithm was based on changes in motor scores, adverse events, and the need for reprogramming.

**Results:**

The mean (±standard deviation) dystonia motor score decreased by 73 ± 24% at 3 years and 63 ± 38% at 5 years for contacts that exhibited acute improvement of dystonia (*n* = 17) during the monopolar review. Contacts without acute benefit improved by 58 ± 30% at 3 years (*n* = 63) and 53 ± 31% at 5 years (*n* = 59). Interestingly, acute worsening or induction of dystonia/dyskinesia (*n* = 9) correlated significantly with improvement after 3 years, but not 5 years.

**Conclusions:**

Monopolar review helped to detect the best therapeutic contact in approximately 30% of patients exhibiting acute modulation of dystonic symptoms. Acute improvement, as well as worsening of dystonia, predicted a good long-term outcome, while induction of phosphenes did not correlate with outcome.

**Trial registration:**

ClinicalTrials.gov NCT00142259.

## Background

Primary dystonia comprises a heterogeneous group of incurable, idiopathic movement disorders with involuntary muscle contractions leading to twisting, repetitive movements and abnormal postures [1, 6]. Oral drug therapy, using combinations of antidopaminergic, anticholinergic, and muscle-relaxing drugs, is often unsatisfactory [5, 6, 13]. If the mainstay treatment – selective peripheral deafferentation by local injection of botulinum toxin – is not feasible or fails, patients are left with severe motor disability and social stigma [2, 6]. For these medically-intractable forms of dystonia, bilateral deep brain stimulation (DBS) of the internal globus pallidus (GPi) is now an established treatment alternative [13]. Previous trials have shown GPi-DBS to be relatively safe and effective, with a favorable benefit-to-risk ratio that is maintained in the long term [3, 6, 11, 13].

The reported benefits from GPi-DBS include a 50–80% reduction in dystonia motor symptoms [3, 6, 11, 13]. Significant improvements in pain, activities of daily living, and quality of life have also been reported after pallidal DBS [13]. Outcomes critically depend on stimulation of a subregion of the GPi [4, 9, 10], which requires accurate positioning of the stimulating lead and selection of appropriate stimulation parameters. However, unlike in Parkinson’s disease, where acute clinical-response testing helps to guide electrode placement intraoperatively and the selection of stimulation settings postoperatively, clinical responses to DBS in dystonia are often delayed, sometimes by days or weeks, which poses a particular challenge in tailoring the therapy. Moreover, indirect guidance of stimulation by adverse effects can be misleading, because muscle contractions from capsular stimulation may be difficult to distinguish from dystonic cramps. Current recommendations for programming DBS in dystonia are pragmatic recommendations based on expert opinion, rather than clinical evidence [8, 12]. In a previous multicenter study on the efficacy and safety of GPi-DBS for patients with generalized or segmental dystonia [6, 13], we standardized stimulation settings and introduced a programming algorithm (PA) for selecting the active electrodes based on an acute monopolar review session. Patients were followed prospectively for up to 5 years within the trial, which allowed us to validate our PA retrospectively according to the clinical evolution and programming history of each patient. Additionally, we tried to identify acute clinical features, which could serve as predictors of the long-term response to stimulation settings determined by this algorithm. We present the results of this evaluation here.

## Methods

The original trial was a double-blind, sham-controlled study for 3 months, followed by an open-label extension for up to 5 years, including 40 patients with pharmacologically-intractable, primary generalized or segmental dystonia [6]. At implantation, the patients were aged 14–75 years, with a disease duration prior to DBS of at least 5 years. All patients received an implanted device for DBS (Kinetra™, Medtronic Inc., Minneapolis, USA) between 2002 and 2004, with stimulation electrodes targeted at the ventro-postero-medial part of the GPi. For localization of the GPi intraoperative microelectrode recordings were used in 29 patients, post-operative MRI in 27.

The algorithm to determine the active stimulating electrode was defined in the study protocol. Within the first week of implantation, a monopolar review of all electrodes of the quadripolar lead (Medtronic 3387 or 3389) was performed. With the IPG as anode (+) and stimulation frequency and pulse width kept constant at 130 Hz and 120 μs, respectively, each electrode, beginning with the most distal one, was stimulated as monopolar cathode (−) with an increasing amplitude to a maximum of 6 V for 60–90 s, as long as no acute adverse effects were elicited. Induction of beneficial effects (e.g. reduction of dystonia, subjective tension or pain) or adverse effects (e.g. increased dystonia, dysesthesia, visual field disturbances, tetanic contractions) were documented. If beneficial effects were noted, the respective electrode was chosen for long-term stimulation. Otherwise, an electrode that elicited phosphenes at an amplitude above 3 V was selected. If phosphenes were induced below 3 V, the next proximal electrode was chosen. At the time of planning the study (around 2000), imaging and image fusion techniques to reconstruct the individual electrode positions postoperatively were not readily available, neither were volume of tissue activated (VTA) models established. Acute clinical response testing was used to establish the anatomical position of the electrodes and based on expert consensus, we considered a phosphene threshold below 3 V as indicating a proximity to the optic tract, indicating a contact location below the ventral border of the GPi. Testing was suspended above 6 V, because higher settings were deemed to be inappropriate for chronic stimulation without causing adverse effects.

**Fig. 1:**
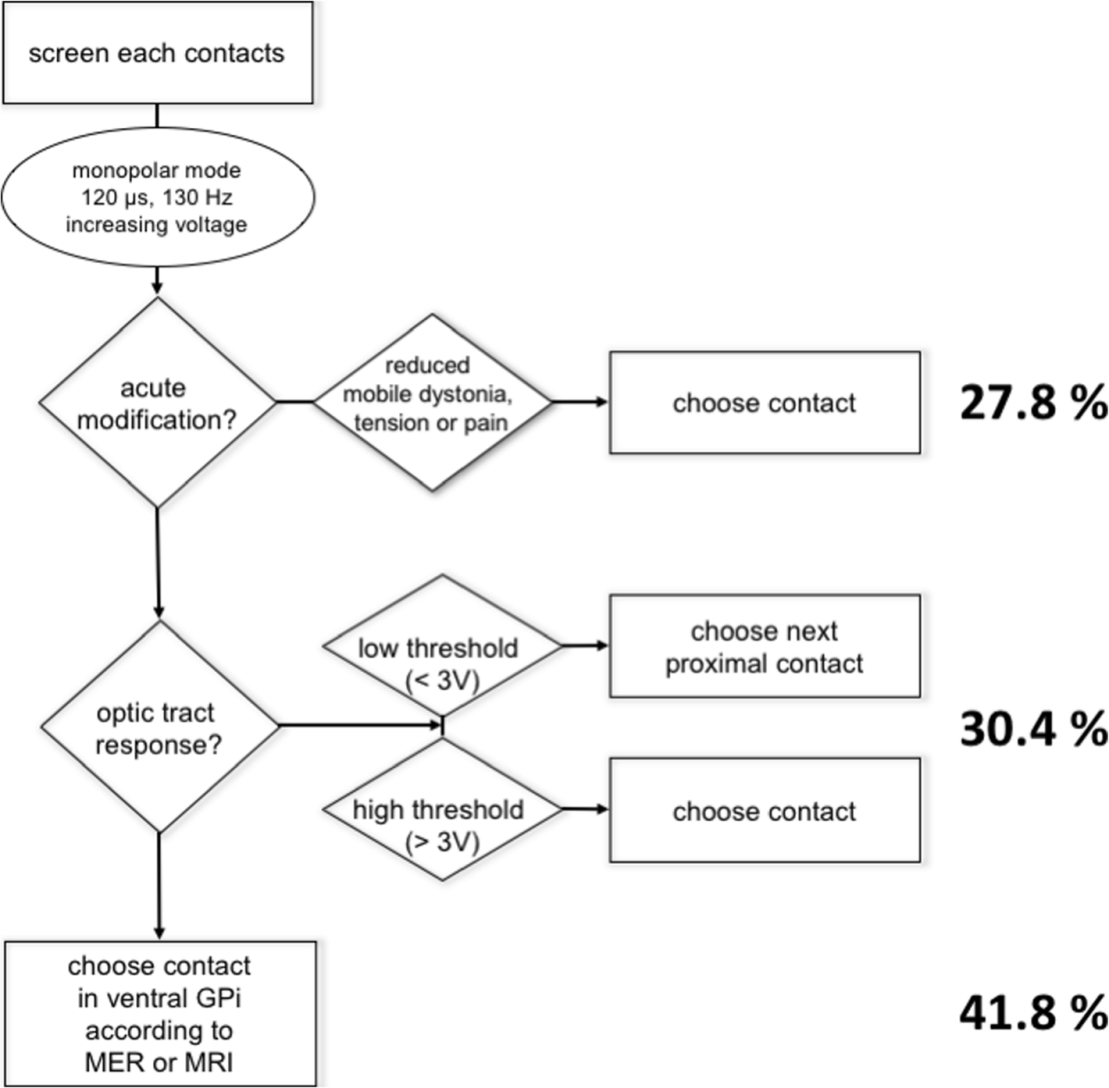
Flow chart describing the basic procedure of the original study protocol for configuring the device during the monopolar review session. Numbers depicted on the right give the percentage of electrode contacts chosen using this criterion at the beginning of our study

If neither beneficial effects nor optic tract response (= phosphenes) could be elicited through any electrode of the lead, the most distal electrode presumed to be located in the ventral GPi, based on intraoperative microelectrode recordings (MER) and/or pre- and postoperative magnetic resonance imaging (MRI), was activated (anatomic choice). Corresponding to the monopolar review, stimulation frequency and pulse width were initially set to 130 Hz and 120 μs. In the neurostimulation group, amplitude was set 0.5 V below the threshold of eliciting adverse effects from the start of the monopolar review, or up to 6 V if no side effects were elicited (Fig. 1).

DBS was started at this amplitude directly after the monopolar review in the stimulation and 3 months later in the sham-stimulation group.

As long as no intolerable adverse effects developed under chronic stimulation, the electrode and amplitude, which was selected based on the PA had to be kept constant for at least 3 months. After 3 months the programmer was allowed to make any adjustment based on his personal experience in case the observed effect was unsatisfactory or adverse effects were observed.

Thirty-eight patients from the original study agreed to participate in the study extension. Three-year-follow-up data were available from 31 patients and five-year data from 32 patients [13]. The efficacy of the PA was evaluated based on the results in these patients. Clinical efficacy was measured by the proportional change in the Burke-Fahn-Marsden Dystonia Rating Scale (BFMDRS) motor score from baseline to the study visit. A decrease of BFMDRS of more than 50% was judged as good, from 25 to 50% as moderate and less than 25% as poor outcome.

Moreover, frequency, threshold, and type of acute stimulation-induced effects and their distribution across electrodes during the monopolar review were evaluated. The predictive value of these features on clinical outcomes was also analyzed (JMP version 13.2.0, SAS Institute Inc., North Carolina, USA). Where appropriate, results are presented as mean ± standard deviation.

## Results

### Criteria for contact selection and compliance to the PA

A total of 27.8% of active contacts were chosen because of acute improvement in dystonic symptoms during the monopolar review. Another 30.4% were chosen because of elicitation of phosphenes. The majority of contacts (41.8%) were selected anatomically (based on nuclear boundaries determined by intraoperative MER and/or MRI), since neither acute effects nor phosphenes could be elicited.

During the initial 6 to 9 months study period, 85% of the electrode configurations remained compliant with the PA; 67% were still compliant at the three- and five-year follow-up. We considered the configuration still compliant if another electrode was added (double monopolar configuration) adjacent to the original one.

The method of choice for the primary contact selection did not have a significant impact on maintenance of the stimulated contacts, i.e. whether based on anatomical aspects (50%), optic-tract response (23%), or acute stimulation benefit (27%). However, there is evidence for a trend towards higher compliance with contacts chosen on an anatomical basis.

### Clinical outcome

At the five-year follow-up, the mean improvement in the BFMDRS score was 57.6 ± 32% if the electrode selection was compliant with the algorithm (*n* = 44 contacts) vs. 53 ± 34% if there was a deviation (*n* = 36 contacts). This difference was not significant.

Electrodes exhibiting acute improvement of dystonia during the monopolar review led to an average decrease in the motor score of 73 ± 24% after 3 years (*n* = 17 contacts) and 63 ± 38% after 5 years (*n* = 21 contacts). This compared to 58 ± 30% after 3 years (*n* = 63 contacts) and 53 ± 31% after 5 years (*n* = 59 contacts) in patients without an acute antidystonic effect. The difference was significant after 3 years (*p* < 0.05), but not after 5 years.

#### Other predictors of clinical outcome

**Fig. 2:**
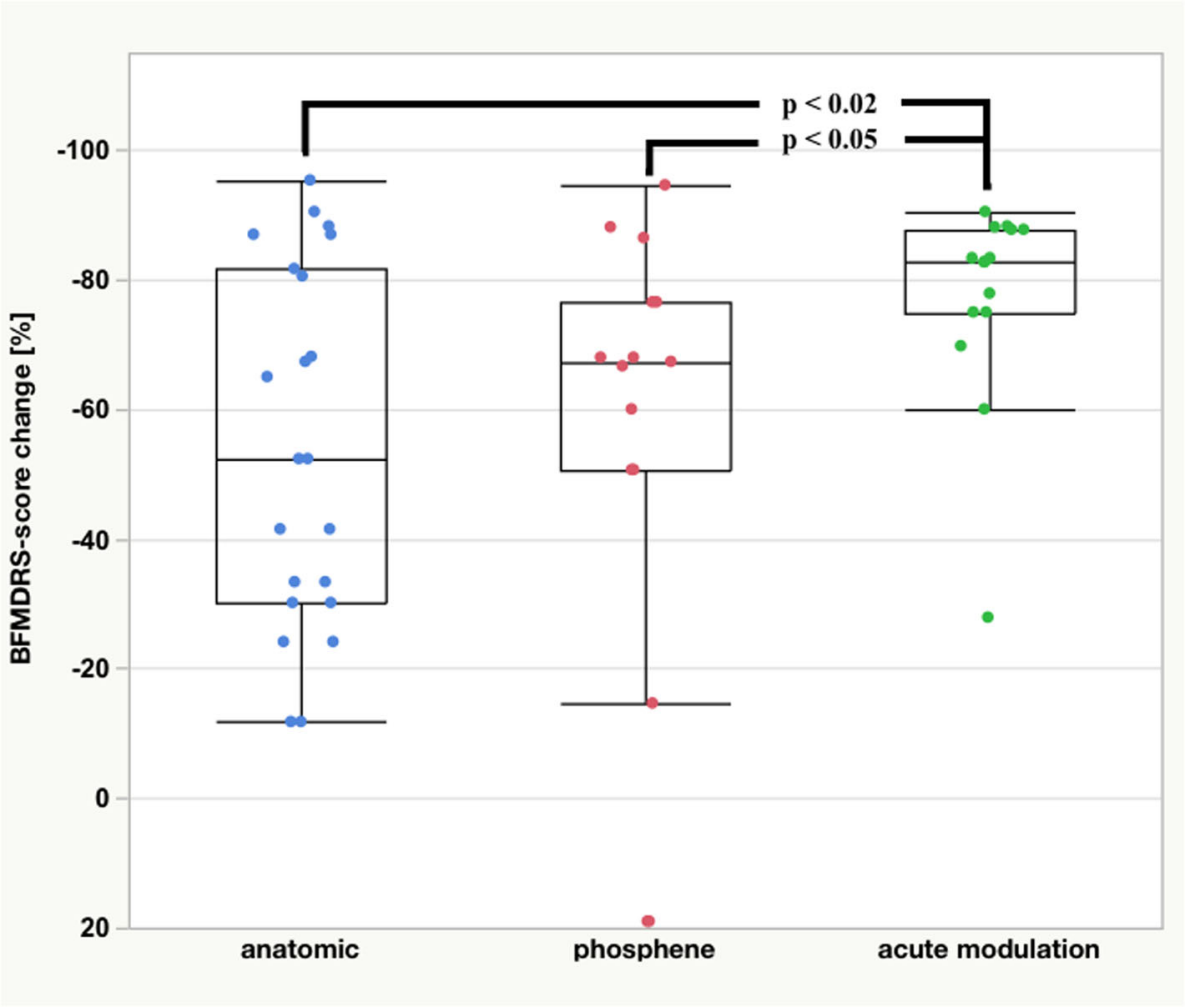
Reduction in the Burke-Fahn-Marsden Dystonia Rating Scale (BFMDRS) motor score after 3 years, depending on choice criteria for contact selection (Wilcoxon multiple

Interestingly, electrodes that evoked acute dyskinesia or worsening of dystonia without inducing capsular side effects (*n* = 9 electrodes, *n* = 9 patients) during the monopolar review were associated with significantly better reduction in the motor score (78 ± 11% after 3 years) compared to 59 ± 31% for all other electrodes (*n* = 71, *p* < 0.005). Electrodes selected based on this kind of acute modulation of dystonia showed significant better response on the BFMDRS after 3 years (77 ± 16%, *n* = 15, *p* < 0.05 for both) compared to those selected anatomically (55 ± 27%; *n* = 23) or based on the optic-tract response (55 ± 36%; *n* = 15) (Fig. 2). After 5 years (*n* = 12 electrodes due to reprogramming), the outcome was still more favorable with a BFMDRS improvement of 61 ± 32% for electrodes with acute modulation of dystonia vs. 54 ± 33% in the remaining, but no longer significant.

Dysarthria was another acute stimulation-induced adverse effect during the monopolar review that was associated with a significant better outcome at 3 years (76 ± 18% vs. 58 ± 18% reduction in the motor score; *n* = 11 vs. 69; *p* < 0.05 Wilcoxon) and a favorable, but non-significant outcome at 5 years (68 ± 22% vs. 53 ± 34%). Other acute adverse effects reported during the monopolar review, such as dysesthesia, visual sensations, nausea, tetanic muscle contractions, or subjective changes in tension and pain in the dystonic body region, had no bearing on the long-term motor benefits.

Contact selections based on any form of acute modulation of dystonic symptoms (improvement as well as worsening or induction of dyskinesias) showed significantly better improvement of motor outcome with a mean decrease in motor score of 77 ± 16% (*n* = 15) after 3 years compared to contacts eliciting phosphenes (55 ± 36%, *n* = 15, *p* < 0.05) or selected on anatomical grounds (55 ± 27%, *n* = 23, *p* < 0.05) (Fig. 2).

Acute effects predicting a good long-term outcome occurred mostly when the second lowest contact of the quadripolar electrode was stimulated. Dysesthesias and capsular side effects were evenly distributed across the contacts and therefore non-localizing.

#### Changes in stimulation during long-term follow-up

A change in the active contact was clinically initiated in 19.6% (*n* = 31) of electrodes after 6 months and in 15.8% at 3 years and 5 years (both *n* = 25) for two reasons: (1) trying to improve efficacy in poor or non-responders (27 electrodes at 6 months, 17 at 3 years, and 18 at 5 years); (2) trying to control stimulation-induced adverse effects (four electrodes at 6 months, seven at 3 years, and seven at 5 years). The most common specific reasons for the stimulation changes documented in the case report forms were “worsening of dystonic symptoms” (*n* = 9) and dysarthria (*n* = 7).

## Discussion

To our knowledge, this is the first study to examine a predefined programming algorithm for pallidal neurostimulation in dystonia. Another unique feature of this study is the long-follow up of 5 years within the framework of a controlled multicenter trial.

When the trial was designed, the ventro-postero-medial segment of the GPi was considered the target region for DBS electrode implantation, based on published case series and experiences from the pallidotomy era. Various studies have since confirmed this area to provide the best overall clinical benefit for pallidal neurostimulation [4, 9, 10]. However, refining the stimulated area by postoperative programming remains challenging, because most studies have observed delayed clinical responses in dystonia, often days or weeks after initiating stimulation.

Here, we show that a standardized monopolar review session helps to detect acute improvements in dystonia in almost one-third of electrodes tested. Stimulation of these contacts was associated with significantly better long-term outcomes than with any other programming choices. Hence, the time spent on a monopolar review session (about 60–90 min for two quadripolar leads) may be well invested in dystonia, even if a smaller proportion of patients can be programmed based on immediate beneficial feedback compared to Parkinson’s disease.

Another third of the electrodes was selected based on stimulation-induced visual phenomena (perception of light/phosphenes) according to our algorithm. This criterion was chosen because this stimulation effect indicates proximity of the electrode to the optic tract, which runs a few millimeters below the target region of the GPi. We found no difference in clinical outcome between the electrodes chosen by visual stimulation effects and those selected on anatomical grounds. Hence, eliciting visual phosphenes provides rough confirmation of anatomically correct positioning of the electrode, but cannot be regarded as a predictive marker for an excellent clinical response [7, 8].

Interestingly, we found that not only acute improvements in dystonia, but also stimulation-induced worsening of dystonia or induction of dyskinesia were associated with an above average outcome. Therefore, any modulation of dystonia during a monopolar review should be regarded as predictive of long-term efficacy; patients can be encouraged to tolerate transient worsening of their condition with the perspective of an excellent outcome.

Among the other acute adverse effects elicited during the monopolar review, only dysarthria predicted a better outcome at 5 years. This is remarkable, because dysarthria is considered an intolerable adverse effect of pallidal stimulation and programming strives to minimize the risk. However, our finding indicates that the cortico-bulbar fibers causing dysarthria may run within the internal capsule in close proximity to the antidystonic “sweet spot” within the GPi and can therefore guide the selection of an optimal electrode, if a sufficient adverse effect threshold is respected.

## Conclusion

In summary, the observations made in this study have the potential to change the clinical approach to programming DBS for dystonia. In a monopolar review, clinicians should search for any acute change in dystonia, either improvement or induction of hyperkinesia, which is predictive for an excellent long-term outcome. These acute modulations of dystonic symptoms were significant better outcome predictor than phosphene, which so far were interpreted as a good outcome predictor, due to the close proximity of the optic tract and ventral internal pallidum. Paradoxically, a low threshold for dysarthria may also indicate an electrode that should be stimulated for optimal outcome below the adverse effect threshold. If none of these acute effects is observed, then programming should be guided anatomically. Recent advances in image processing and software technology provide options for fast and accurate electrode location within the individual anatomical space, which may facilitate this anatomical selection in the future.

## Data Availability

ADK, FS and JV had full access to all data, and take responsibility for the integrity of the data and the accuracy of the data analysis presented in the manuscript.

## Abbreviations

BFMDRS: Burke-Fahn-Marsden Dystonia Rating Scale
DBS: Deep brain stimulation
GPi: Internal globus pallidus
MER: Microelectrode recordings
MRI: Magnetic resonance imaging
PA: Programming algorithm
